# Mitochondrial-Immune Dysfunction in MS: Therapeutic Potential of EV-Mediated Transfer

**DOI:** 10.1007/s10571-026-01716-8

**Published:** 2026-04-17

**Authors:** Parsa Navazi, Mohammad Fereidouni, Nafiseh Erfanian

**Affiliations:** 1 Student Research Committee, Birjand University of Medical Sciences, Birjand, Iran; 2 Cellular and Molecular Research Center, Birjand University of Medical Sciences, Birjand, Iran

**Keywords:** Multiple sclerosis, Mitochondria, Extracellular vesicles, Neuroinflammation, Neurodegeneration

## Abstract

Multiple sclerosis (MS) is a chronic immune-mediated disorder of the central nervous system. In this disease, mitochondrial dysfunction contributes to neurodegeneration, axonal loss, and progressive disability. Extracellular vesicle (EV)-mediated mitochondrial transfer has emerged as a promising cell-free strategy to restore mitochondrial homeostasis and modulate immune responses within the CNS. Preclinical studies, particularly in experimental autoimmune encephalomyelitis models, demonstrate that EV-based delivery of mitochondrial cargo improves cellular bioenergetics. In parallel, this approach reduces oxidative stress and neuroinflammation while supporting remyelination and neuroprotection. This review summarizes the mechanistic rationale, current preclinical evidence, and future translational perspectives of EV-mediated mitochondrial therapy in MS.

**Graphical Abstract:**

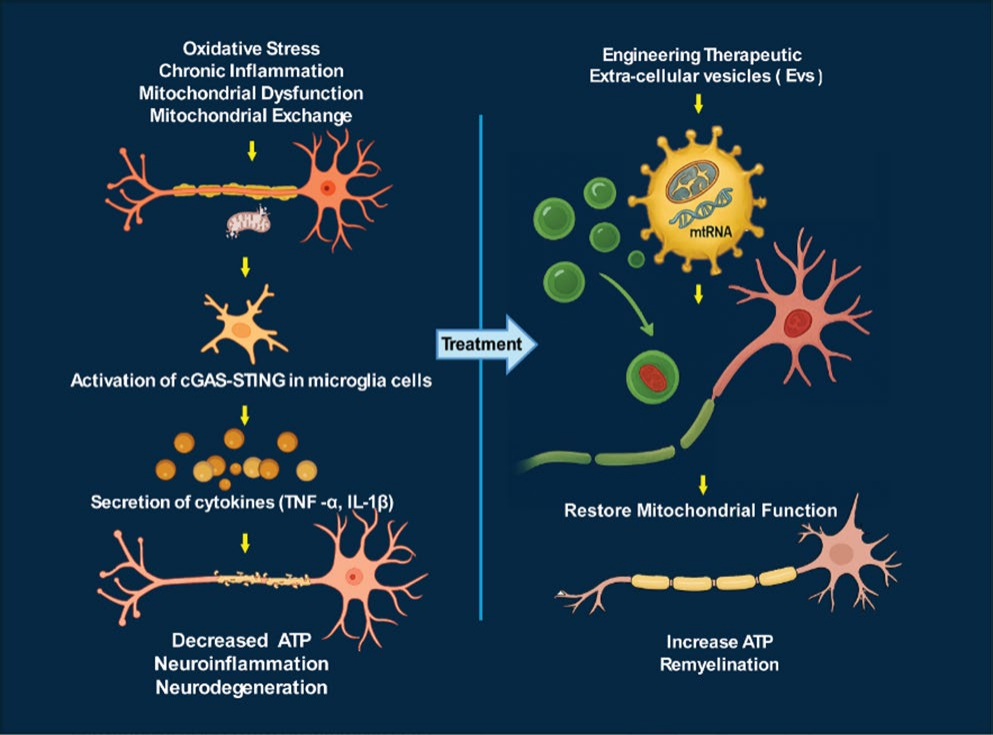

Schematic illustration showing the dual role of neuronal–microglial interaction under pathological and therapeutic conditions. (Left) Neuronal injury caused by oxidative stress, reduced ATP production, and reactive oxygen species (ROS) results in mitochondrial damage and the release of mtDNA, which is taken up by microglia, leading to microglial activation and neuroinflammation. (Right) Under therapeutic conditions, extracellular vesicle (EV)-mediated transfer of mitochondrial components or protective factors restores mitochondrial function, reduces neuroinflammation, and promotes neuronal recovery

**Supplementary Information:**

The online version contains supplementary material available at 10.1007/s10571-026-01716-8.

## Introduction

Multiple sclerosis (MS) is a chronic immune-mediated neuroinflammatory disorder that affects over 2.8 million people worldwide, leading to substantial disability and socioeconomic burden. It is characterized by demyelination, neurodegeneration, and disruption of the blood–brain barrier (BBB). MS predominantly affects the brain, spinal cord, optic nerves, and cerebellum (Compston and Coles 2008). The disease arises from a complex interplay between dysregulated immune responses and progressive neuronal injury, ultimately leading to demyelinating plaques and chronic neurological decline (Androdias et al. 2025). Despite the availability of disease-modifying therapies (DMTs), current treatments mainly focus on immunosuppression and fail to effectively halt or reverse neurodegeneration. Their efficacy in progressive MS is limited, and long-term use is often associated with adverse effects, poor compliance, teratogenicity, and the development of neutralizing antibodies (Hemmer and Mühlau 2017). Most importantly, existing DMTs lack direct neuroprotective mechanisms, emphasizing the urgent need for innovative dual-action strategies that target both immune dysregulation and mitochondrial dysfunction (Bunai et al. 2018).

Mitochondrial impairment is a central hallmark of MS. It includes reduced mitochondrial content, mtDNA deletions, oxidative stress, and impaired oxidative phosphorylation. These abnormalities are observed in demyelinated spinal cord regions as well as in normal-appearing white matter (Atkinson et al. 2025). These alterations result in diminished ATP production, excessive reactive oxygen species (ROS) generation, and neuronal apoptosis, collectively driving irreversible axonal damage (Mahad et al. 2008; Sadeghian et al. 2016; Barcelos et al. 2019; Liu et al. 2025).

To counteract this metabolic failure, the central nervous system naturally employs intercellular mitochondrial transfer as a compensatory mechanism, whereby healthy mitochondria are delivered from supportive cells to stressed neurons. Harnessing this endogenous process, engineered extracellular vesicles represent a promising therapeutic strategy for delivering functional mitochondrial cargo to restore energy homeostasis and mitigate neurodegeneration in MS (Geng et al. 2023).

In this process, healthy mitochondria are delivered from supportive cells to metabolically stressed neurons, restoring energy homeostasis and reducing oxidative stress (Baudo 2024). Mitochondria can be exchanged via tunneling nanotubes (TNTs), extracellular vesicles, gap junctions, or transient cell fusion, contributing to tissue repair, immune modulation, and energy recovery (Lee et al. 2023; Scheiblich et al. 2024).

The therapeutic potential of leveraging this mechanism has attracted increasing attention. If spontaneous mitochondrial transfer can rescue energy-deprived neurons, engineered extracellular vesicles capable of delivering functional mitochondrial cargo represent a promising strategy to mitigate neurodegeneration in MS. Compared to cell-based therapies, extracellular vesicles (EVs) offer several advantages, including low immunogenicity, the ability to cross the BBB, minimal tumorigenicity, and reduced risk of unintended engraftment, while allowing scalable production and engineering for cell-specific targeting (Zhang et al. 2024).

This review synthesizes current evidence on EV-mediated mitochondrial therapy in MS, encompassing three complementary strategies: delivering intact mitochondria, transferring mitochondrial components such as mtDNA and proteins, and engineering EVs with mitochondrial-targeting ligands to enhance delivery specificity. By integrating mechanistic insights, preclinical validations, and future translational perspectives, this article highlights how harnessing mitochondrial transfer through EVs may represent a paradigm-shifting approach for MS and other neurodegenerative or neuroinflammatory diseases. All abbreviations used in this manuscript are defined in Supplementary Table 1.

## Mitochondrial Dysfunction at the Immune-Neural Interface

Mitochondrial dysfunction is a central pathological hallmark of MS, directly contributing to neuroinflammation, axonal degeneration, and disease progression (Mishra et al. 2025). Post-mortem and imaging studies have revealed decreased mitochondrial content, mtDNA deletions, oxidative stress, and impaired oxidative phosphorylation in demyelinated spinal cord regions (Dutta et al. 2006) and even in normal-appearing white matter (NAWM) (Hostenbach et al. 2020; Brier et al. 2025). These abnormalities lead to reduced ATP production, elevated reactive oxygen species (ROS), and neuronal apoptosis, ultimately resulting in irreversible axonal damage. Importantly, mitochondrial failure occurs early and diffusely, underscoring its critical role in MS pathogenesis (Obrador et al. 2021).

While mitochondrial impairment primarily affects neurons, leading to energy failure and axonal loss, its consequences in immune cells such as microglia and infiltrating lymphocytes via the release of mitochondrial damage-associated molecular patterns (mtDAMPs) and subsequent innate immune activation create a vicious cycle that sustains chronic inflammation and hinders neuronal recovery (Mishra et al. 2025).

Beyond intrinsic mitochondrial defects, recent studies suggest that dynamic interactions at the immune–neural interface may also drive mitochondrial loss. One proposed mechanism, intercellular mitochondrial exchange, is discussed in the following section.

### Intercellular Mitochondrial Exchange by Hyperactive Immune Cells

Intercellular mitochondrial exchange transfers mitochondria or their components between CNS cells. It is increasingly seen as a contributor to neuroinflammation and energy depletion in MS. In pathological settings, hyperactive immune cells, including activated microglia and infiltrating T cells, may acquire mitochondria from neurons or glial cells, thereby exacerbating neuronal energy failure (Nakano et al. 2024).

In the CNS, mitochondria can traverse between neurons, glial cells, immune cells, and even tumor cells via multiple well-characterized routes, including tunneling nanotubes (TNTs), extracellular vesicles, receptor-mediated endocytosis, gap junction channels (e.g., Cx43), and direct intercellular contacts (Pinto et al. 2021; Geng et al. 2023). These transfers are orchestrated by mitochondrial trafficking machinery, such as Miro1/Miro2 and TRAK/Milton complexes, which tether mitochondria to cytoskeletal tracks for intercellular transport (Modi et al. 2019). While these mechanisms are well-established in cancer and ischemic injury, direct evidence in MS remains elusive, although their occurrence in other pathologies supports the plausibility of a similar process in neuroinflammatory conditions.

Several processes may facilitate this exchange in MS:

- Tunneling nanotubes (TNTs) and extracellular vesicles: Bidirectional conduits for the trafficking of intact mitochondria or mitochondrial components between neurons, glial cells, and immune cells, supporting intercellular metabolic communication (D’Anca et al. 2021; Iorio et al. 2024).
- Metabolic reprogramming of immune cells: Activated microglia and infiltrating T cells may increasingly depend on exogenous mitochondria to sustain heightened energy demands, predisposing them to acquire mitochondria from neighboring neural cells (Artusa et al. 2025).
- Mitochondrial stress and byproduct release: Oxidative stress and iron dysregulation in neurons can lead to the extrusion of mitochondria or mitochondrial fragments, which are then captured by immune cells. This process depletes the neuronal mitochondrial pool and reinforces a self-perpetuating cycle of neuroinflammation (Tavassolifar et al. 2020; Wang et al. 2023a).

Although these mechanisms are mechanistically plausible, confirmation in MS will require advanced in vivo studies, including live-cell imaging, mitochondrial labeling, and single-cell tracking in experimental autoimmune encephalomyelitis (EAE) and post-mortem tissue. Collectively, intercellular mitochondrial exchange represents a potential driver of energy failure, persistent neuroinflammation, and neurodegeneration in MS, and may emerge as a novel therapeutic target through modulation of mitochondrial dynamics.

### Mitophagy and Mitochondrial Quality Control Failure

Mitophagy, a selective form of autophagy, is fundamental to mitochondrial quality control, ensuring the timely removal of damaged or dysfunctional mitochondria. This process maintains cellular energy homeostasis and prevents excessive oxidative stress, which is particularly crucial in highly energy-dependent cells such as neurons and glial cells. In MS, accumulating evidence indicates that impaired mitophagy is a pivotal driver of disease progression rather than a secondary byproduct of neuroinflammation (Kadowaki et al. 2025). When mitophagy is disrupted, damaged mitochondria persist within the cell and release mitochondrial byproducts, including mtDNA and ROS, into the cytosol (Lin et al. 2019). These byproducts act as mtDAMPs that activate innate immune sensors, notably the cyclic GMP-AMP synthase stimulator of interferon genes (cGAS–STING) pathway (Zamiri et al. 2023). Subsequent activation of this pathway triggers type I interferon (IFN-I) production and pro-inflammatory cytokine release, perpetuating chronic neuroinflammation (Decout et al. 2021). The molecular basis of defective mitophagy in MS involves both genetic and environmental contributors. Variants in key mitophagy regulators, such as NDP52 (CALCOCO2), reduce mitophagic efficiency and enhance pro-inflammatory cytokine production in immune cells, amplifying neuroinflammation (Di Rita et al. 2021). Concurrently, chronic oxidative stress and sustained ROS generation disrupt autophagic flux, further impairing the PINK1/Parkin-dependent mitophagy pathway. Elevated expression of mitophagy markers, such as Parkin, during active disease phases reflects an insufficient compensatory attempt to clear dysfunctional mitochondria (Castellazzi et al. 2019). Collectively, mitophagy failure leads to a self-reinforcing cycle of mitochondrial accumulation, metabolic collapse, and inflammatory amplification. This cascade accelerates demyelination, axonal degeneration, and irreversible neurodegeneration, particularly in progressive MS.

Impaired mitophagy not only perpetuates inflammatory signaling via mtDAMP release (detailed in Sect. "cGAS–STING Pathway: Linking Mitochondrial Damage to Chronic Neuroinflammation") but also exerts primary metabolic effects that directly contribute to demyelination. Accumulation of dysfunctional mitochondria reduces oxidative phosphorylation efficiency, leading to ATP depletion and disrupted energy homeostasis. Oligodendrocytes, which have exceptionally high energetic demands for myelin synthesis, maintenance, and repair, are particularly vulnerable to this metabolic stress. Insufficient ATP compromises lipid and protein synthesis required for myelin sheath formation, impairs oligodendrocyte maturation and process extension, and hinders remyelination in demyelinated lesions (López-Muguruza and Matute 2023; Soung et al. 2025).

Furthermore, energy failure in oligodendrocytes disrupts metabolic coupling with axons, including lactate shuttling via monocarboxylate transporter 1 (MCT1), a critical mechanism for providing trophic support to axons. This exacerbates axonal energy deficits, promotes axonal damage, and contributes to a vicious cycle of demyelination and neurodegeneration (Fünfschilling et al., 2012; López-Muguruza and Matute 2023). Preclinical evidence supports this link: enhancing mitophagy (e.g., through inhibition of negative regulators like USP30) improves mitochondrial bioenergetics in oligodendrocyte lineage cells, accelerates their differentiation, and promotes remyelination in models of demyelination (Soung et al. 2025).

**Fig. 1:**
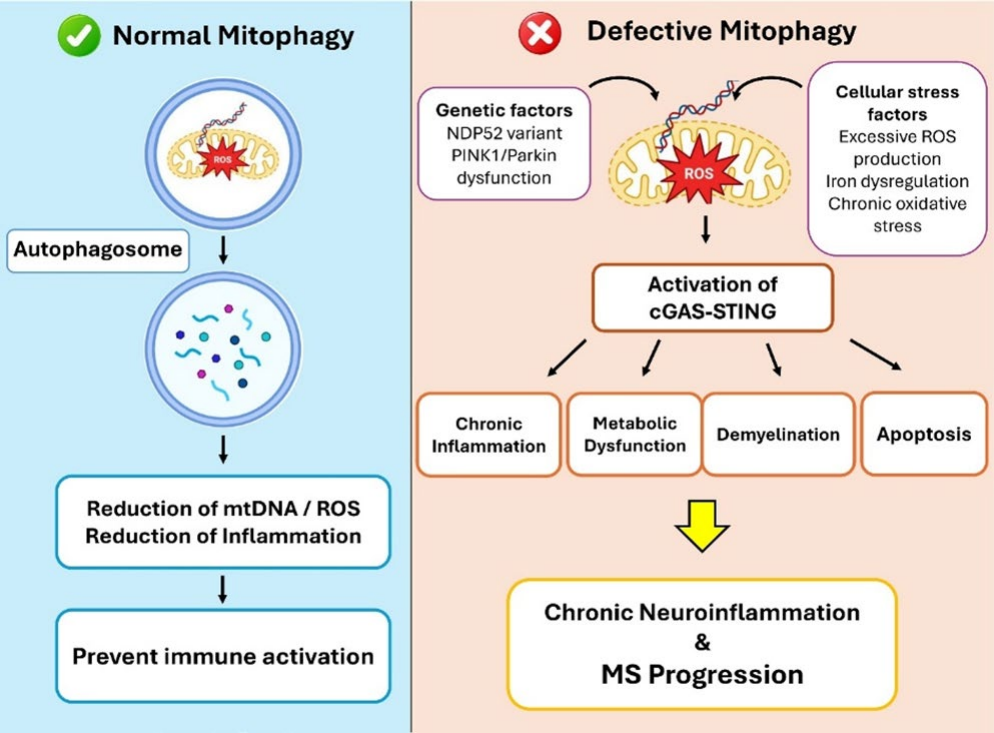
Impaired mitophagy and its contribution to neuroinflammation and MS progression. Under normal conditions, mitophagy removes damaged mitochondria to maintain mitochondrial homeostasis and prevent excessive oxidative stress. In MS, impaired mitophagy leads to the accumulation of dysfunctional mitochondria, resulting in the release of mtDNA and ROS into the cytosol and inducing pro-inflammatory cytokine production. This chronic inflammatory signaling contributes to demyelination, axonal damage, and disease progression, particularly in progressive MS. *MS* multiple sclerosis, *mtDNA* mitochondrial DNA, *ROS* reactive oxygen species All schematic illustrations were created using Adobe Illustrator version 2025 (Adobe Inc.)

Thus, mitophagy serves not only as a mitochondrial quality control mechanism but also as a critical modulator of neuroimmune homeostasis. Targeting mitophagy pathways offers a promising therapeutic avenue to limit neurodegeneration and restore cellular resilience in MS (Fig. 1).

### cGAS–STING Pathway: Linking Mitochondrial Damage to Chronic Neuroinflammation

Building on mitophagy failure and mtDAMP release (Sect. "Mitophagy and Mitochondrial Quality Control Failure"), the cGAS–STING pathway serves as a key innate immune sensor linking mitochondrial dysfunction to chronic neuroinflammation in MS. When mtDNA enters the cytosol, cGAS synthesizes cyclic GMP-AMP (cGAMP), which activates STING on the endoplasmic reticulum. STING then initiates TANK-binding kinase 1 (TBK1) and Interferon regulatory factor 3/7 (IRF3/7) signaling, leading to interferon type I (IFN-I) production, primarily IFN-β (Mosallanejad and Kagan 2022; Maimaiti et al. 2024). IFN-I promotes the transcription of interferon-stimulated genes (ISGs) such as CXCL10, ISG15, and MX1, which contribute to an antiviral and pro-inflammatory milieu (Huang et al. 2025a). Prolonged IFN-I signaling enhances antigen presentation via upregulation of MHC class I on neural and glial cells and sustains microglial activation. These processes collectively drive chronic immune activation and neuroinflammation, hallmark features of progressive MS (Ignarro et al. 2022; Perdaens and Van Pesch 2022). In parallel, STING activation triggers the NF-κB and MAPK pathways, while also promoting lysosomal destabilization and potassium efflux, both of which facilitate the assembly of the NOD-like receptor family, pyrin domain-containing 3 (NLRP3) inflammasome (Gaidt et al. 2017; Liu et al. 2024). Importantly, once activated, the inflammasome recruits caspase-1, leading to the maturation and release of IL-1β and IL-18 and inducing pyroptosis, a lytic form of programmed inflammatory cell death (Cui et al. 2022; Malhotra et al. 2023). The resulting release of pro-inflammatory cytokines and cytosolic contents further activates STING, reinforcing the self-perpetuating inflammatory cycle (Dubowsky et al. 2023).

**Fig. 2:**
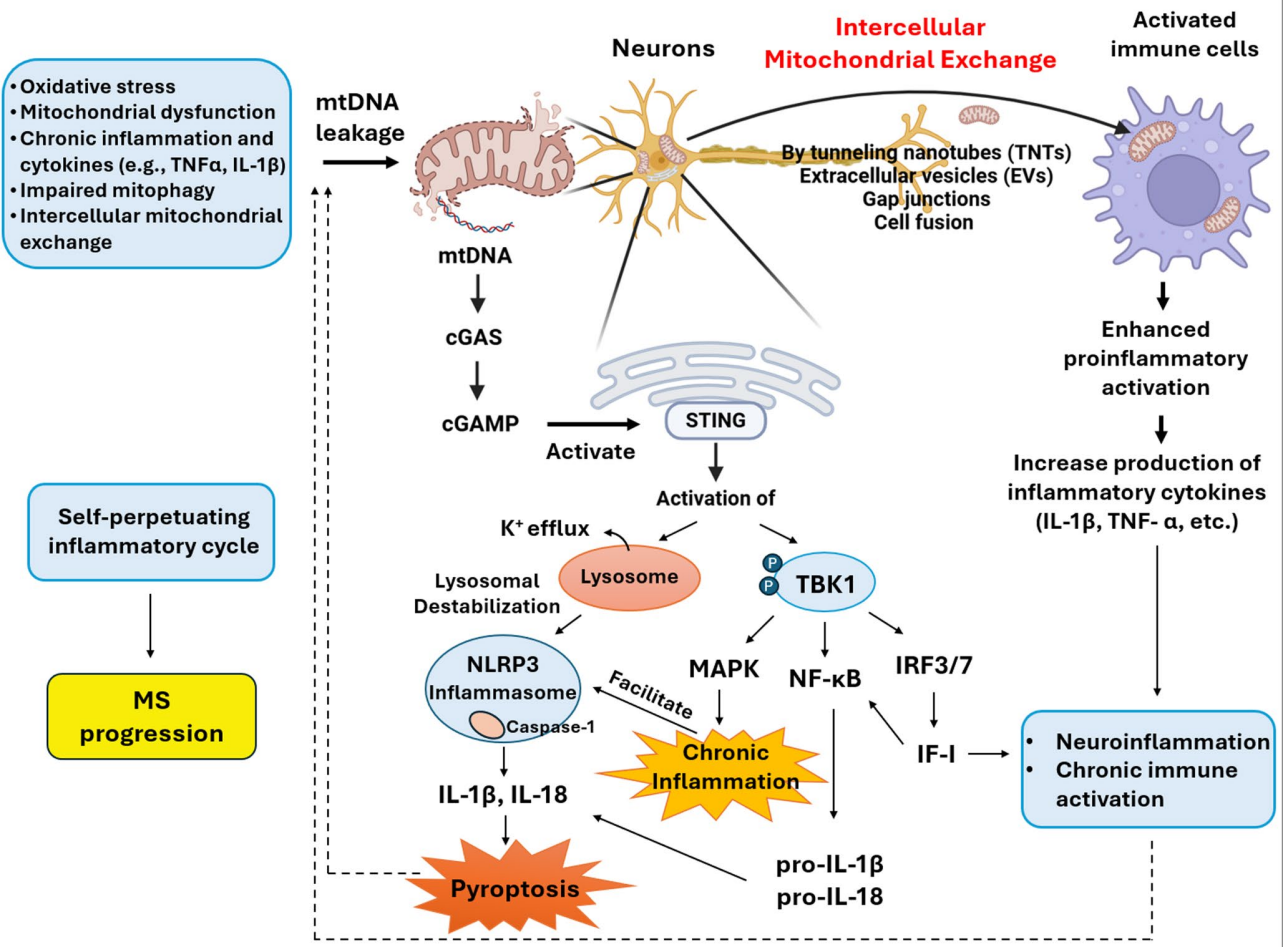
Schematic representation of mitochondrial dysfunction in neurons contributing to neuroinflammation. Damaged mitochondria release mtDNA into the cytosol, which activates the cGAS–STING signaling pathway and triggers IRF3/7 phosphorylation via TBK1, ultimately leading to the production of proinflammatory cytokines. *mtDNA* mitochondrial DNA, *cGAS–STING* cyclic GMP–AMP synthase – Stimulator of Interferon Genes pathway, *IRF3/7* Interferon regulatory factor 3/7, *TBK1* TANK-binding kinase 1 All schematic illustrations were created using Adobe Illustrator version 2025 (Adobe Inc.)

This pathological loop is active in multiple CNS cell types, including neurons (where it directly contributes to energy failure and axonal degeneration) and microglia (where it sustains chronic activation and neuroinflammation), as well as CNS-infiltrating myeloid cells. Although mitochondrial impairment is a primary driver of metabolic defects in neurons, the resulting mtDNA release and cGAS–STING activation in microglia and infiltrating immune cells (including lymphocytes) amplify and perpetuate the chronic neuroinflammatory cycle characteristic of progressive MS. Its role is believed to be especially prominent in progressive forms of MS, where neurodegeneration predominates and current immunomodulatory therapies have limited efficacy (Liu et al. 2022; Li et al. 2024). Notably, several approved MS treatments, such as interferon-beta (IFN-β) (Feng et al. 2019) and fingolimod (Hartung et al. 2023), have been shown to reduce mtDNA leakage or modulate STING activity, thereby potentially disrupting this damaging inflammatory circuit (Fig. 2).

### Biomarkers of Mitochondrial Failure in MS

Mitochondrial failure in MS is closely associated with disease activity, progression, and neuroinflammation. Therefore, identifying biomarkers that reflect mitochondrial damage can be instrumental in diagnosis, disease monitoring, and therapeutic evaluation. One such biomarker is cell-free mtDNA in cerebrospinal fluid (CSF), which is elevated during active phases of MS and significantly decreases following aggressive therapies such as autologous hematopoietic stem cell transplantation (aHSCT) (Fyfe 2024). Similarly, peripheral blood mtDNA copy number (mtDNA-CN) has been found to fluctuate with disease activity, typically reduced during remission and elevated during relapses, making it a potential tool for tracking disease fluctuations (Sedky et al. 2023).

Other emerging markers include growth differentiation factor 15 (GDF-15) and fibroblast growth factor 21 (FGF-21), both of which are stress-responsive cytokines associated with mitochondrial stress. Notably, elevated levels of GDF-15 have been correlated with MS severity (Nohara et al. 2019). Although not specific to mitochondria, neurofilament light chain (NF-L) is a widely used biomarker of axonal damage and has been linked to mitochondrial failure due to its downstream association with neurodegeneration (Ghezzi and Neuteboom 2023). Metabolic profiling has also been explored, including assessments of lactate, pyruvate, creatine kinase, and amino acid patterns. However, findings related to these markers have shown variability across MS populations and require further validation (Blagov et al. 2022). In addition to biochemical markers, neuroimaging techniques such as positron emission tomography (PET) have proven useful for visualizing mitochondrial dysfunction in vivo. PET using [18 F]-fluorodeoxyglucose ([18 F]-FDG) reveals patterns of hypometabolism in both lesion areas and normal-appearing white matter, indicative of mitochondrial energy deficits (Faria Dde et al. 2014). Moreover, PET imaging targeting the 18-kDa translocator protein (TSPO), which is upregulated in activated microglia and macrophages, serves as a surrogate for neuroinflammation and indirectly reflects the interplay between inflammation and mitochondrial failure. Elevated TSPO signals have been shown to correlate with active disease states in MS patients (Poutiainen et al. 2016; Madani Neishaboori et al. 2024). These molecular and imaging biomarkers offer valuable tools for detecting mitochondrial involvement in MS and may facilitate patient stratification for mitochondrial-targeted therapies.

## Engineering Strategies for EV-Mediated Mitochondrial Therapy

Recent advances in nanomedicine have positioned extracellular vesicles, including exosomes and microvesicles, as promising delivery platforms for neurodegenerative disease therapies (Tao and Gao 2024). In MS, where mitochondrial failure is pivotal in sustaining chronic neuroinflammation and driving neurodegeneration, restoring mitochondrial health has emerged as a promising therapeutic strategy. Leveraging the intrinsic ability of EVs to transfer bioactive molecules between cells and cross the blood–brain barrier (BBB), these vesicles provide a unique platform for delivering mitochondrial components directly to metabolically compromised neural and immune cells within the central nervous system (CNS) (Zhao et al. 2025).

Extracellular vesicles are nanoscale, membrane-bound particles naturally secreted by almost all cell types, including neurons, glial cells, and immune cells. They encompass multiple subtypes, primarily exosomes, microvesicles, and apoptotic bodies, each differing in size, biogenesis, and cargo composition. EVs can traverse the blood–brain barrier (BBB) and efficiently transport bioactive molecules such as proteins, mRNAs, microRNAs, and mitochondrial components, making them highly attractive candidates for CNS-targeted therapy, including in MS (Pusic et al. 2014; Zonouz et al. 2025). By engineering EVs to carry functional transfer of mitochondrial DNA (mtDNA), mitochondrial proteins, and in some cases even intact mitochondria, innovative strategies aim to restore neuronal bioenergetics, reduce oxidative stress, and ultimately halt or reverse neurodegeneration in MS (Picone and Nuzzo 2022; Mozafari et al. 2025).

### Loading Intact Mitochondria into EVs

Loading intact, functional mitochondria into EVs offers a direct route to restore cellular bioenergetics in injured tissues. In pioneering studies, human mesenchymal stromal cells (MSCs) were shown to release EVs containing viable mitochondria that could be internalized by recipient cells leading to enhanced oxidative phosphorylation and ATP production (Thomas et al. 2022). Similarly, cardiomyocyte-derived mitochondrial extracellular vesicles (mitoEVs) isolated from human induced pluripotent stem cell (iPSC)-derived cardiomyocytes improved myocardial energetics and reduced infarct size in a mouse model of myocardial infarction (Ikeda et al. 2021). Efforts to enrich mitochondrial cargo have employed strategies such as pharmacological or genetic activation of mitochondrial biogenesis in donor cells. For example, activation of PGC‑1α via resveratrol in endothelial cells increased mitochondrial load in secreted EVs, creating mitoEVs that significantly elevated ATP levels in recipient brain endothelial cells (Dave et al. 2022). Beyond MSCs and cardiomyocytes, neuroglial interactions also demonstrate the physiological relevance of intact mitochondrial transfer. In a murine stroke model, astrocytes released functional mitochondria that were internalized by neighboring neurons via a calcium- and CD38-dependent mechanism, promoting neuronal survival and recovery after ischemic injury (Hayakawa et al. 2016). Consistent with these findings, neural stem cells (NSCs) have also been shown to deliver functional mitochondria via EVs, restoring bioenergetics in recipient cells and ameliorating neuroinflammation in models of multiple sclerosis (Peruzzotti-Jametti et al. 2021). This neuroprotective effect highlights that EV-mediated mitochondrial delivery can not only enhance energy metabolism but also modulate CNS repair mechanisms. Despite these advances, several challenges remain, including standardizing mitochondrial encapsulation methods, preserving mitochondrial viability during EV isolation, and achieving efficient and targeted delivery to CNS cells. Addressing these issues is essential for successful translation of mitoEV-based therapy to multiple sclerosis and other neurodegenerative disorders. The experimental validation of these strategies in cellular and animal models is summarized in Sect. "Preclinical Evidence and Validation".

### Transfer of Mitochondrial Components (mtDNA and Proteins)

EVs can transfer not only intact mitochondria but also critical mitochondrial subcomponents, including mtDNA, mitochondrial proteins, and small mitochondrial fragments. These cargo elements contribute to restoring bioenergetic function, regulating cellular signaling, and modulating inflammatory responses, while avoiding the complexity and potential immunogenicity associated with whole-mitochondria transfer (Welsh et al. 2024). mtDNA encodes essential components of the oxidative phosphorylation (OXPHOS) system, including 13 polypeptides critical for ATP production, as well as ribosomal and transfer RNAs (Rambani et al. 2022). Delivery of mtDNA via EVs can restore mitochondrial gene expression in recipient cells (Peruzzotti-Jametti et al. 2021). Studies have shown that mtDNA damage in MS contributes to impaired OXPHOS, reduced ATP levels, and neuronal apoptosis, which can be partially reversed by delivering functional mtDNA to affected neurons (Qin et al. 2024).

Mitochondrial proteins, such as cytochrome c oxidase, ATP synthase subunits, and antioxidant enzymes like superoxide dismutase (SOD), are also critical for maintaining mitochondrial homeostasis. EVs can deliver these proteins to recipient cells, enhancing mitochondrial repair and reducing oxidative stress (Perrier et al. 2025). For example, transfer of mitochondrial transcription factor A (TFAM), a key regulator of mtDNA replication and transcription, stabilizes mitochondrial function in preclinical neurodegeneration models (Oka et al. 2016). Preclinical studies in experimental autoimmune encephalomyelitis (EAE), the primary animal model of MS, have demonstrated the therapeutic potential of EV-mediated mitochondrial component transfer (Mozafari et al. 2025). Studies in EAE highlight the reduced effectiveness of axonal mitochondrial mobilization, suggesting a role for EVs in restoring bioenergetics. This is supported by analogous studies in neurodegenerative models (Peruzzotti-Jametti et al. 2021).

The delivery of specific mitochondrial proteins, such as superoxide dismutases, via EVs has been investigated primarily in models of oxidative stress and neuroinflammation (D’Souza et al. 2021; Qi et al. 2021; Chen et al. 2025b). Although direct evidence in EAE remains limited, studies in related neuroinflammatory and ischemic models have shown that EV-mediated transfer of antioxidant enzymes can attenuate ROS accumulation and suppress activation of the NLRP3 inflammasome, a central driver of chronic inflammation in MS (Ayyubova et al. 2023). Collectively, these findings highlight the therapeutic promise of mitochondrial component transfer. This approach can simultaneously address bioenergetic failure and aberrant inflammatory cascades in MS. It also supports the rationale for designing mitoEVs (Chen et al. 2025b).

Compared with whole-mitochondria delivery, EV-mediated transfer of mitochondrial components offers several advantages. mtDNA and proteins can be isolated or synthesized and loaded into EVs using established techniques such as electroporation or sonication. These components are more stable than intact mitochondria, which are prone to damage during EV loading and systemic delivery (Danilushkina et al. 2023; Grignano et al. 2025).

### Candidate Cell Sources for Engineered EVs in MS Therapy

The selection of appropriate source cells for engineered EVs production is a critical factor in optimizing therapeutic outcomes in MS. Various cell types, both native to the central nervous system and of mesenchymal origin, offer distinct advantages due to their innate regenerative, immunomodulatory, or neuroprotective properties (Table 1). Mesenchymal Stem Cells (MSCs) remain the most extensively studied source of therapeutic EVs. MSC-derived EVs have demonstrated strong immunomodulatory, anti-inflammatory, and neuroprotective effects in MS models. Notably, they shift microglial phenotypes from pro-inflammatory to anti-inflammatory states and support remyelination through delivery of trophic factors and regulatory RNAs (Zhang et al. 2022). Umbilical cord–derived MSC exosomes (UMSC-Exos) modified with TAxI peptides have shown enhanced CNS targeting and therapeutic efficacy in EAE models, reducing inflammation and promoting microglial polarization (Wang et al. 2023b). In addition, IFN-γ–preconditioned MSCs produce exosomes enriched with Indoleamine 2,3-dioxygenase (IDO) and immunoregulatory miRNAs, promoting tolerogenic immune responses and reducing EAE clinical severity (Riazifar et al. 2019).

Oligodendrocyte Precursor Cells (OPCs) are increasingly recognized for their ability to stimulate remyelination and provide trophic support to axons. These vesicles contain cargo that can modulate T-cell polarization from Th1 to Th2 and promote gliosis attenuation, especially when combined with neural stem cell therapy (Santos et al. 2024). EVs secreted by neurons and glial cells in the CNS have been shown to transport protective cargo and contribute to neuroprotection and neurogenesis, highlighting their intrinsic therapeutic potential in MS (Hasaniani et al. 2024). While less explored for engineering, they represent biologically relevant delivery vehicles due to their homing to CNS tissue and compatibility with neural microenvironments.

Engineered Cell Lines such as HEK293T have been utilized for high-efficiency delivery of therapeutic agents like brain-derived neurotrophic factor (BDNF). These engineered EVs have shown success in inducing OPC differentiation, enhancing remyelination, and improving motor function in demyelination models via intranasal administration (Zhai et al. 2022).

Finally, genetically or pharmacologically modified cells can be used to generate EVs enriched in mitochondrial content, enabling mitochondrial replacement or repair strategies (Chen et al. 2025a; Lou et al. 2025).

**Table 1:** Candidate cell sources for engineered EVs in MS therapy

Cell source	Advantages	Therapeutic role in MS	Reference
Mesenchymal stem cells (bone marrow, umbilical cord)	Strong immunomodulation, cross-BBB capacity, scalable production	Shift microglia to an anti-inflammatory state, reduce neuroinflammation, and support remyelination	Riazifar et al. (2019) Zhang et al. (2022) Wang et al. (2023b)
Oligodendrocyte precursor cells (OPCs)	Promote myelination, provide trophic and axonal support	Enhance remyelination, modulate T-cell polarization (Th1 → Th2), reduce gliosis	Santos et al. (2024)
Neurons and glial cells (astrocytes, microglia, oligodendrocytes)	Natural CNS-derived EV profile, intrinsic homing to neural tissue	Deliver protective and neurogenic cargo, support neuroprotection and neurogenesis	Hasaniani et al. (2024)
Engineered cell lines (e.g., HEK293T)	Easily engineered, high yield, reproducible	Deliver neurotrophic factors (e.g., BDNF), promote OPC differentiation, and improve motor recovery	Zhai et al. (2022)
Genetically or pharmacologically modified cells	Customizable EV cargo, mitochondrial enrichment possible	Enable mitochondrial repair/replacement, enhance remyelination, improve neuroregeneration	Chen et al. (2025a); Lou et al. (2025)

*BBB* Blood–Brain Barrier, *OPCs* Oligodendrocyte Precursor Cells, *CNS* Central Nervous System, *MSCs* Mesenchymal Stem Cells, *BDNF* Brain-Derived Neurotrophic Factor, *HEK293T* Human Embryonic Kidney 293T cells

### Mechanisms of Mitochondrial Loading into EVs

Functional mitochondrial loading into EVs can be achieved through several optimized approaches. Extrusion through nanoporous membranes applies mechanical shear forces that mimic natural vesicle formation, facilitating cargo encapsulation. This approach has been shown to improve the loading efficiency of proteins and nucleic acids into EVs. While direct evidence for intact mitochondrial loading is limited, extrusion represents a promising strategy for increasing EV cargo content (Park et al. 2025). Sonication-assisted loading is a method for incorporating cargo into EVs. It uses high-frequency ultrasound to transiently disrupt the EV membrane. This allows incorporation of mitochondrial components, such as mtDNA, respiratory proteins, and small mitochondrial fragments (Lamichhane et al. 2016; Colja et al. 2023). This method works well for nucleic acids and proteins, but has limitations for intact mitochondria due to their size and fragility. Metabolic modulation of donor cells, such as inducing metabolic stress or inhibiting lysosomal function (e.g., with chloroquine), can increase the secretion of mitochondria-rich EVs. This approach leverages endogenous mitochondrial quality control mechanisms, enhancing the release of EVs containing intact or partial mitochondrial components (Phinney et al. 2015; Sansone et al. 2017). Genetic and peptide engineering approaches allow donor cells to be modified to overexpress mitochondrial-anchoring proteins (e.g., Miro1) or targeting peptides to selectively enrich EVs with mitochondrial components. Similarly, pharmacological stimulation of mitochondrial biogenesis (e.g., via PGC-1α activation using resveratrol) increases mitochondrial content in EVs (Dave et al. 2022). Recent studies have demonstrated dual-targeted EVs for delivering mitochondrial circRNAs, such as circMTCO2. These EVs enable specific delivery to neuronal mitochondria, resulting in precise functional rescue in disease models (Shi et al. 2025).

**Fig. 3:**
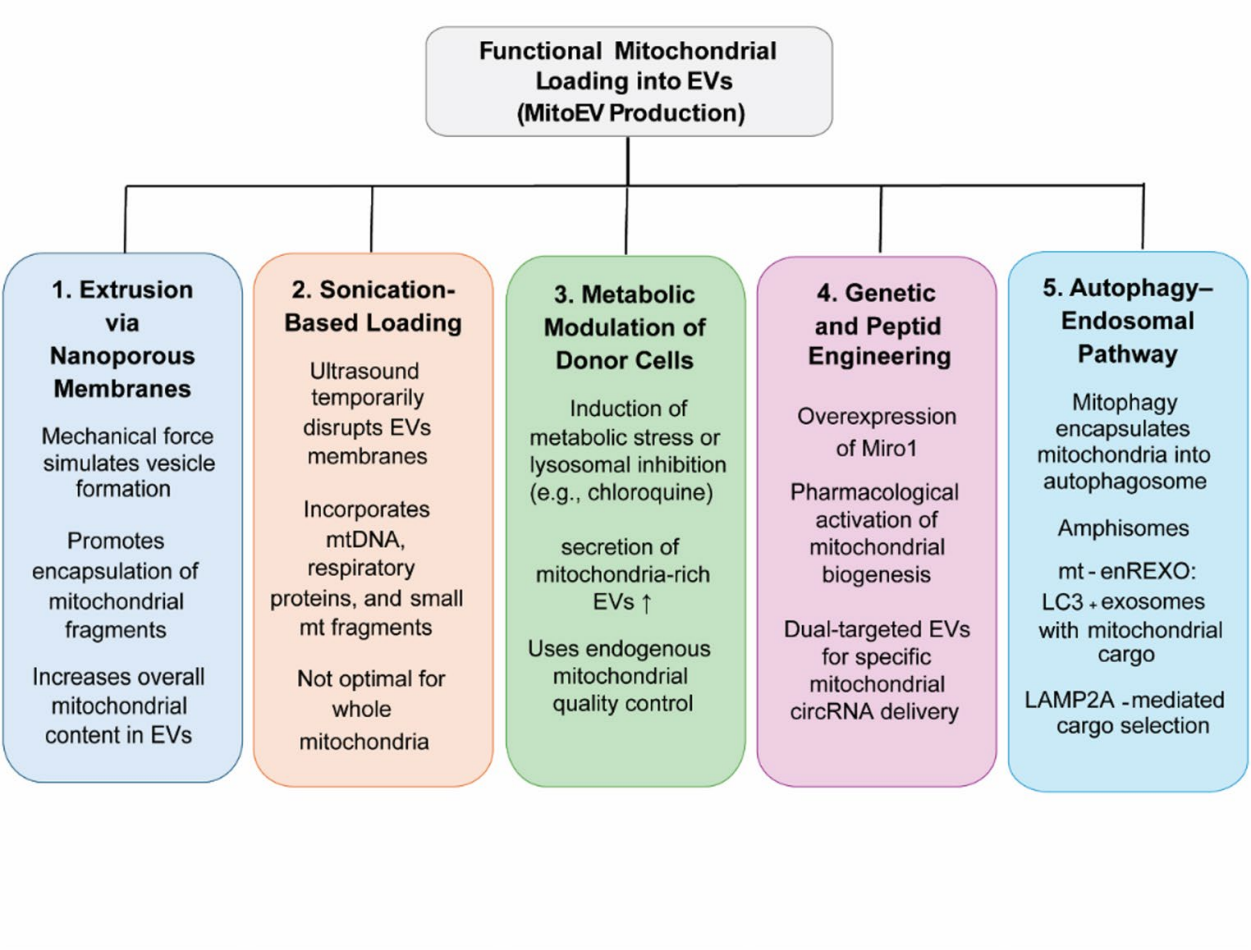
Schematic overview of strategies for loading functional mitochondria into extracellular vesicles (mitoEVs), including: extrusion through nanoporous membranes, sonication-assisted membrane permeabilization, metabolic modulation of donor cells, mitochondrial biogenesis stimulation via genetic engineering, and autophagy endosomal pathways. These approaches aim to enhance mitochondrial cargo packaging and improve the therapeutic efficacy of mitoEVs All schematic illustrations were created using Adobe Illustrator version 2025 (Adobe Inc.)

Autophagy- and endosome-mediated loading occur during mitophagy. In this process, damaged mitochondria are encapsulated into autophagosomes. These autophagosomes can fuse with multivesicular bodies (MVBs) to incorporate mitochondrial components into EVs. Key proteins such as LAMP2A facilitate selective loading of mitochondrial proteins, while ceramide-mediated lipid raft pathways provide an ESCRT-independent alternative (Ferreira et al. 2022; Jahangiri et al. 2022). Despite their potential, natural pathways have limited efficiency for clinical translation. This motivates the development of synthetic EV mimetics to improve scalability and mitochondrial cargo delivery. However, these advances must be balanced against risks and barriers, discussed in the next section. These opportunities, however, must be carefully balanced against potential risks and translational barriers, which will be discussed in the following section (Li et al. 2021) (Fig. 3).

### Preclinical Evidence and Validation

Preclinical studies, primarily in experimental autoimmune encephalomyelitis (EAE) and demyelination models, have validated the therapeutic potential of EV-mediated mitochondrial transfer. Key findings include restoration of bioenergetics, reduction in oxidative stress and neuroinflammation, and promotion of remyelination. These in vitro and in vivo results support the proposed strategies (intact mitochondrial loading and component delivery) while underscoring the need for improved targeting and scalability (Table 2).

**Table 2:** Summary of preclinical studies on EV-mediated mitochondrial transfer

Study	Model (in vitro/in vivo)	Cell source/EV type	Key findings	Reference
Hayakawa et al. (2016)	In vitro (cell co-culture) and in vivo (stroke model)	Astrocyte-derived EVs	Transfer of functional mitochondria to stressed neurons improved survival and recovery	Hayakawa et al. (2016)
Oka et al. (2016)	In vitro (neurodegeneration models)	EVs delivering TFAM	Stabilized mitochondrial function, partial rescue of OXPHOS, and ATP levels	Oka et al. (2016)
Peruzzotti-Jametti et al. (2021)	In vivo (EAE) and in vitro (recipient cells)	Neural stem cell-derived EVs	Restored bioenergetics, ameliorated neuroinflammation, and functional mitochondrial delivery	Peruzzotti-Jametti et al. (2021)
D’Souza et al. (2021)	In vitro (oxygen-glucose deprivation/ischemia models)	Brain endothelial cell-derived microvesicles	Transfer of polarized mitochondria to BECs and neurons; 100–200-fold ATP increase; improved mitochondrial function and endothelial survival in ischemia; microvesicles better than exosomes	D’Souza et al. (2021)
Thomas et al. (2022)	In vitro (MSC EV transfer)	MSC-derived mitoEVs	Enhanced mitochondrial respiration, ATP production, and reduced oxidative stress	Thomas et al. (2022)
Dave et al. (2022)	In vitro (brain endothelial cells)	Endothelial cell-derived mitoEVs	Improved ATP levels, reduced oxidative stress	Dave et al. (2022)
Zhang et al. (2022)	In vivo (demyelinating CNS)	MSC-derived EVs	Reduced neuroinflammation, supported remyelination through microglial modulation	Zhang et al. (2022)
Wang et al. (2023a, 2023b)	In vivo (EAE)	Chimeric CNS-targeting MSC EVs	Ameliorated disease severity, reduced neuroinflammation, and preserved axonal integrity	Wang et al. (2023b)
Santos et al. (2024)	In vivo (EAE)	OPC-derived EVs (combined with NSCs)	Attenuated inflammation, modulated microglial activation, promoted recovery	Santos et al. (2024)
Zhang et al. (2024)	In vivo (neurodegenerative models)	Engineered activated neutrophil EVs	Crossed BBB, neuroprotective effects without transplantation risks	Zhang et al. (2024)
Chen et al. (2025a, 2025b)	In vitro (neuroinflammatory/ischemic models)	MSC-derived EVs with mitochondrial cargo	Addressed bioenergetic failure and inflammation	Chen et al. (2025a)
Mozafari et al. (2025)	In vivo (EAE, demyelination models)	Various EV types	Enhanced remyelination, neuroprotective effects	Mozafari et al. (2025)

*EVs* extracellular vesicles, *MSCs* mesenchymal stem cells, *OPCs* oligodendrocyte precursor cells, *NSCs* neural stem cells, *CNS* central nervous system, *EAE* experimental autoimmune encephalomyelitis, *BECs* brain endothelial cells, *BBB* blood–brain barrier, *TFAM* mitochondrial transcription factor A, *OXPHOS* oxidative phosphorylation, *ROS* reactive oxygen species, *NLRP3* NOD-, LRR- and pyrin domain–containing protein 3 inflammasome, *mitoEVs* mitochondria-containing extracellular vesicles

## Challenges and Future Directions

mitoEVs offer a promising therapeutic avenue for MS, yet several barriers must be addressed before clinical translation. Key challenges include scalable EV production, efficient encapsulation of intact mitochondria, and vesicle stability during storage and distribution (Kim et al. 2024). Targeted delivery to the CNS is difficult. Selective uptake by neurons, oligodendrocytes, or immune cells often requires surface modifications. Safety is also critical. Mitochondrial components, especially mtDNA, may trigger inflammation. This requires rigorous toxicology and biodistribution studies (Clemente-Suárez et al. 2023). Emerging strategies hold potential to overcome these limitations. CRISPR-based engineering could optimize donor cell mitochondria and improve packaging efficiency, while stimuli-responsive or “smart” EVs are being developed to achieve controlled release in response to pathological signals (Whitley and Cai 2023; Akyuz et al. 2024). In the context of MS, such innovations may enhance mitochondrial resilience and support remyelination. Together, these advances position mitoEVs as a potentially transformative, yet technically demanding, therapeutic modality (Wagstaff et al. 2024).

A major hurdle is the chronic neuroinflammatory and oxidative microenvironment in MS. This creates a hostile milieu that threatens therapeutic durability. Persistent oxidative and nitrosative stress impairs EV uptake and biodistribution. This occurs through altered BBB permeability (Mozafari et al. 2025), immune clearance, and reduced target-cell receptivity in metabolically decoupled oligodendrocytes and neurons (Hu et al. 2025). Transferred mitochondria face direct damage. ROS/RNS oxidize lipids, proteins, and mtDNA (Huang et al. 2025b). They cause enzyme inactivation (Ciccocioppo et al. 2019) and mtDAMP release, perpetuating inflammation (Hermann et al. 2024; Huang et al. 2025b). This diverts therapeutic EVs toward inflammatory pathways.

Although direct longitudinal evidence of therapy loss due to this milieu is limited, the mechanistic rationale is clear: transferred mitochondria are vulnerable to rapid impairment in progressive lesions (Huang et al. 2025b). Thus, single-dose interventions are unlikely to sustain bioenergetic rescue or break ongoing vicious cycles. To achieve durable effects, integrated multimodal strategies are required. These include: (1) repeated/sustained EV administration for cumulative replenishment (Mozafari et al. 2025); (2) combined protective cargo (e.g., antioxidant enzymes like superoxide dismutase) to shield mitochondria from oxidative damage (Hu et al. 2025); (3) concurrent immunomodulation with established therapies (e.g., fingolimod/siponimod) to reduce clearance and inflammation (Hermann et al. 2024); and (4) advanced surface modifications for better targeting and resistance to diversion (Wang et al. 2023b). Mitophagy enhancers may add synergy by improving endogenous quality control (Soung et al. 2025). Long-term studies in chronic EAE models and clinical trials are needed to validate these approaches, providing a feasible path toward lasting neuroprotection in progressive MS.

## Conclusion

mitoEVs offer a transformative therapeutic avenue that integrates metabolic rescue, immune modulation, and targeted delivery in MS. Through advancements in engineering, biogenesis control, and precision targeting, these nano-vesicles hold the potential to overcome current limitations in disease-modifying therapies, especially in progressive forms of MS. Significant challenges remain, from scalable production to clinical validation. However, emerging innovations such as CRISPR-enhanced exosomes and biomarker-guided stratification may soon bridge the gap from bench to bedside. Harnessing the full therapeutic capacity of mitoEVs could redefine the future landscape of neurodegenerative disease management. From a clinical perspective, EV-mediated mitochondrial therapies are likely to follow a stepwise translational path, beginning with advanced preclinical toxicology and biodistribution studies, followed by early-phase clinical trials within the next 5–10 years if manufacturing and safety benchmarks are met. Major regulatory challenges include standardization of EV production, batch-to-batch consistency, long-term safety assessment, and regulatory classification of mitoEVs as advanced biological therapeutics. Addressing these hurdles through harmonized manufacturing protocols and biomarker-guided patient stratification will be essential for clinical adoption. Harnessing the full therapeutic capacity of mitoEVs may ultimately redefine the future landscape of MS and neurodegenerative disease management (Burnouf et al. 2019; Verma and Arora 2025).

## Supplementary Information

Below is the link to the electronic supplementary material.

Supplementary file1 (TIF 14 kb)

## Data Availability

No datasets were generated or analysed during the current study.
