# Supplementary material for: Mitochondrial-Immune Dysfunction in MS: Therapeutic Potential of EV-Mediated Transfer
Source: Cell Mol Neurobiol. 2026 Apr 17;46:95. doi: 10.1007/s10571-026-01716-8 (PMC13216381; doi:10.1007/s10571-026-01716-8)
Supplement: Supplementary file 1 — Supplementary file1 (TIF 14 kb) [file 10571_2026_1716_MOESM1_ESM.docx]

Supplementary:

| Abbreviation | Full Name | Definition/Description |
| --- | --- | --- |
| MS | Multiple Sclerosis | Chronic immune-mediated neuroinflammatory disorder of the CNS characterized by demyelination and neurodegeneration. |
| EV | Extracellular Vesicle | Nanoscale membrane-bound particles secreted by cells that transport bioactive molecules (proteins, RNA, mitochondria) between cells. |
| BBB | Blood–Brain Barrier | Selective barrier protecting the CNS from blood-borne substances. |
| BECs | Brain Endothelial Cells | Specialized endothelial cells forming the blood-brain barrier (BBB) and regulating exchange between blood and CNS. |
| OPC | Oligodendrocyte Precursor Cell | Progenitor cells that differentiate into mature oligodendrocytes for myelin production. |
| CNS | Central Nervous System | Brain and spinal cord. |
| mtDNA | Mitochondrial DNA | Circular DNA in mitochondria encoding key components of oxidative phosphorylation. |
| ROS | Reactive Oxygen Species | Highly reactive molecules that can damage cellular components (e.g., lipids, proteins). |
| cGAS–STING | Cyclic GMP–AMP Synthase–Stimulator of Interferon Genes | Innate immune pathway activated by cytosolic DNA (e.g., mtDNA), leading to type I interferon production and inflammation. |
| mtDAMP | Mitochondrial Damage-Associated Molecular Pattern | Mitochondrial byproducts (e.g., mtDNA, ROS) released upon damage that trigger innate immune responses. |
| mitoEV | Mitochondria-loaded Extracellular Vesicle | EVs engineered or naturally containing mitochondrial cargo (intact mitochondria or components) for therapeutic delivery. |
| OXPHOS | Oxidative Phosphorylation | Mitochondrial process for ATP production via the electron transport chain. |
| ATP | Adenosine Triphosphate | Primary energy currency of the cell. |
| EAE | Experimental Autoimmune Encephalomyelitis | Animal model of MS used for preclinical studies. |
| MSC | Mesenchymal Stem Cell | Common source of therapeutic EVs with immunomodulatory and regenerative properties. |
| DMT | Disease-Modifying Therapy | Current treatments for MS that modulate immune responses. |
| IFN-I | Type I Interferon | Cytokines (e.g., IFN-β) involved in antiviral and pro-inflammatory responses. |
| NLRP3 | NOD-like receptor family pyrin domain containing 3 | Inflammasome component activated by mtDAMPs leading to IL-1β/IL-18 release. |
| PGC-1α | Peroxisome Proliferator-Activated Receptor Gamma Coactivator 1-Alpha | Master regulator of mitochondrial biogenesis. |
| MCT1 | Monocarboxylate Transporter 1 | Transporter for lactate shuttling from oligodendrocytes to axons. |
| BDNF | Brain-Derived Neurotrophic Factor | Neurotrophic factor promoting neuronal survival and remyelination. |
